# New insights of aquaporin 5 in the pathogenesis of high altitude pulmonary edema

**DOI:** 10.1186/1746-1596-8-193

**Published:** 2013-11-25

**Authors:** Jun She, Jing Bi, Lin Tong, Yuanlin Song, Chunxue Bai

**Affiliations:** 1 Department of Pulmonary Medicine, Zhongshan Hospital, Fudan University, NO.180 Fenglin Road, Shanghai 200032, China

**Keywords:** Aquaporin 5 (AQP5), High altitude pulmonary edema (HAPE), Inflammation, Wet/dry weight ratio

## Abstract

**Background:**

High altitude pulmonary edema (HAPE) affects individuals and is characterized by alveolar flooding with protein-rich edema as a consequence of blood-gas barrier disruption. In this study, we hypothesized that aquaporin 5 (AQP5) which is one kind of water channels may play a role in preservation of alveolar epithelial barrier integrity in high altitude pulmonary edema (HAPE).

**Methods:**

Therefore, we established a model in Wildtype mice and AQP5 −/− mice were assingned to normoxic rest (NR), hypoxic rest (HR) and hypoxic exercise (HE) group. Mice were produced by training to walk at treadmill for exercising and chamber pressure was reduced to simulate climbing an altitude of 5000 m for 48 hours. Studies using BAL in HAPE mice to demonstrated that edema is caused leakage of albumin proteins and red cells across the alveolarcapillary barrier in the absence of any evidence of inflammation.

**Results:**

In this study, the Lung wet/dry weight ratio and broncholalveolar lavage protein concentrations were slightly increased in HE AQP5 −/− mice compared to wildtype mice. And histologic evidence of hemorrhagic pulmonary edema was distinctly shown in HE group. The lung Evan’s blue permeability of HE group was showed slightly increased compare to the wildtype groups, and HR group was showed a medium situation from normal to HAPE development compared with NR and HE group.

**Conclusions:**

Deletion of AQP5 slightly increased lung edema and lung injury compared to wildtype mice during HAPE development, which suggested that the AQP5 plays an important role in HAPE formation induced by high altitude simulation.

## Background

High-altitude pulmonary edema (HAPE) is an acute potentially fatal disease that occurs as a result of a rapid exposure to high altitude (3000 m above sea level) [1-3]. Due to exposed to altitudes higher than 3,000 m above sea level, the HAPE morbidity rate ranges from 0.4 to 2 [4,5]. Previous studies have shown that HAPE has family-specific, race-specific and individual susceptibility tendencies [6]. After arrived in high altitude, the amount of alveolar fluid depends on its’ dynamic balance between escaping liquid from the pulmonary vasculature and the reabsorption by the alveolar respiratory epithelium [7]. It is believed that high lung water permeability facilitates fluid formation and resolution of pulmonary edema [8]. Therefore, ideal HAPE treatment should protect the integrity of alveolar-capillary membrane and increase the alveolar fluid reabsorption. Unfortunately, the HAPE is a noncardiogenic, hydrostatic lung edema which has been characterized as a non-inflammatory high permeability type leak caused by stress failure [9-11]. To date, about HAPE pathogenesis is not fully understood. Previous evidence suggested that uneven hypoxic pulmonary vasoconstriction, pulmonary capillary damage and increased pulmonary artery pressure play important roles in HAPE pathogenesis [12,13]. The endothelial barrier has been commonly believed as the initial mechanism triggering the development of HAPE [14]. In addition, some previous results showed that endothelial and epithelial stress failure were contribute to HAPE development in a rat model of HAPE morphological studies [15]. So far, there are many difficulties during effective treatments against HAPE, such as alveolar epithelium swelling and epithelium barrier disruption. So the epithelium and the epithelium barrier become the new target of the study in the maintenance of alveolar-capillary integrity and normal alveolar function during HAPE development. The aquaporins (AQP) are a family of membrane water channels, which facilitate water transport across cell membranes in response to osmotic gradients [11-13] and involved in physiological functions including water/salt homeostasis, epidermal hydration and exocrine fluid secretion [16]. Aquaporin 5 (AQP5) is one of the members of AQP which is expressed at apical membrane of alveolar type I cell in the lungs [17]. Some studies were shown that AQP5 facilitates the principal route for osmotically driven water transport between alveolar epithelium and capillary compartments [17,18]. In the airways, though AQP3 and AQP4 facilitate osmotic water transport also, their deletion does not impair fluid absorption [19,20]. In contrast to these, AQP5 deletion significantly reduced alveolar-capillary osmotic water permeability up to 15 fold in mice [17]. And high pulmonary vascular pressures when expose to hypoxia result in transient breakdown of the integrity of the alveolar-capillary barrier, permitting fluid accumulation [21,22]. Therefore, we postulated that AQP5 may be beneficial in hydrostatic lung edema. We hypothesized that AQP5 may play an important role in alveolar epithelial barrier function in formation of lung edema involve fluid transport for HAPE. In this study, we aimed to test whether AQP5 facilitate the integrity of alveolar epithelia barrier and beneficiate for the integrity of alveolar-capillary barrier, using our established mouse model and explored the possible mechanism.

## Materials and methods

### Animals

The AQP5 −/− knockout and wild type adult male mice (weight 18–23 g) were generously provided by Dr. Alan S. Verkman (University of California, San Francisco) and maintained at the Fudan University animal facility. The protocol was approved by the Animal Care Committees of Fudan University. All animals were conducted in accordance with the Guidelines for the Use and Care of Research Animals published by the National Institutes of Health.

### Hypoxia exposure procedure

Nine AQP5 −/− knockout mice participated in this study and were randomly assigned as the NR group (mice exposed to normobaric normoxia at rest), HR group (mice exposed to hypobaric hypoxia at rest) and HE group (mice exposed to hypobaric hypoxia with exercise). In addition, nine wildtype mice were seen as a control in the same grouping method. The research protocol was performed as described previously [15]. Briefly, mice were trained for exercising in treadmill and the chamber pressure was gradually reduced (20 m/sec) to reach a simulate altitude of 5000 m above sea level. The treadmill walking speed was setup to a exercise intensity of 6 m/min. Mice of NR group had never been exposed to normobaric normoxia caused by an independently controlled antechamber used in the hypobaric hypoxic procedures. Hypoxia exposure procedure took about 20–30 minutes interval and animals supplied fresh food and water every 4 hours regularly. All data was collected at a safety simulated altitude of 3800 m above sea level [23].

### Bronchoalveolar lavage (BAL)

Bronchoalveolar lavage (BAL) was selectively performed with the left hemi-lung. The lungs were injected with 0.5 ml 0.9% saline, and then gently retrieved into sterile containers [21] after 2–3 times gentle flushing. The red and white blood cell counts were performed on Sysmex KX-21 (Sysmex Co., Ltd, Kobe, Japan). Total protein and albumin were measured using acid titration and immunoturbidimetry by Automatic Analyzer (HITACHI-7600, Hitachi, Ltd, Lbaraki Japan) respectively [15].

### Lung wet-to-dry weight ratio (W/D)

The trachea were inserted into the mice after tracheotomy in a supine position. The lungs were isolated after chest opening, and the right superior lobe of hemi-lung was exsanguinated and excised. After wet weights were measured on electronic scale (HANGPING FA1104, Shanghai Precision & Scientific Instrument Co., Ltd, China), the lungs were dried in an oven (Shanghai Yuejin Medical Instruments Factory, China) at 56°C for 72 hours in order to get the dry weights. Lung wet-to-dry weight ratio was calculated as described previously [15,24,25].

### Lung histology

The rest of right middle lobe lung was immersed in formalin, embedded in paraffin, sectioned and stained with hematoxylin and eosin for histological studies. The severity of lung injury was inspected by light microscopy (Nikon Eclipse 50i, Nikon Instruments Inc., Tokyo, Japan) [25].

### Alveolar-capillary permeability

The alveolar-capillary permeability was performed with Evans Blue dye (EBD) method [24,26]. Briefly, One third of each group were performed the intravenous injection of EBD (30 mg/kg) for an estimate of alveolar-capillary membrane leakage through the caudal vein which avoiding the influence on conventional results in this study [27]. The lung was separated and incubated at 37°C for 24 h and centrifuged at 5,000 × g for 30 min. The optical density of the supernatant was measured by optical density on the supernatant using spectrophotometry at 620 nm. The concentration of EBD was determined from a standard curve of EBD formamide solutions.

### Statistical analyses

The data were displayed as mean and Standard Error Of Mean (SEM) unless otherwise specified using SAS 6.12 software (SAS Institute Inc., North Carolina, USA). In addition, multiple groups compared were performed by one-way analysis of variance (ANOVA) procedures with Bonferroni test [28]. As well, when the statistical significance was reached (alpha level of *p* ≤0.05) where significant mean differences occurred.

## Results

**Figure 1:**
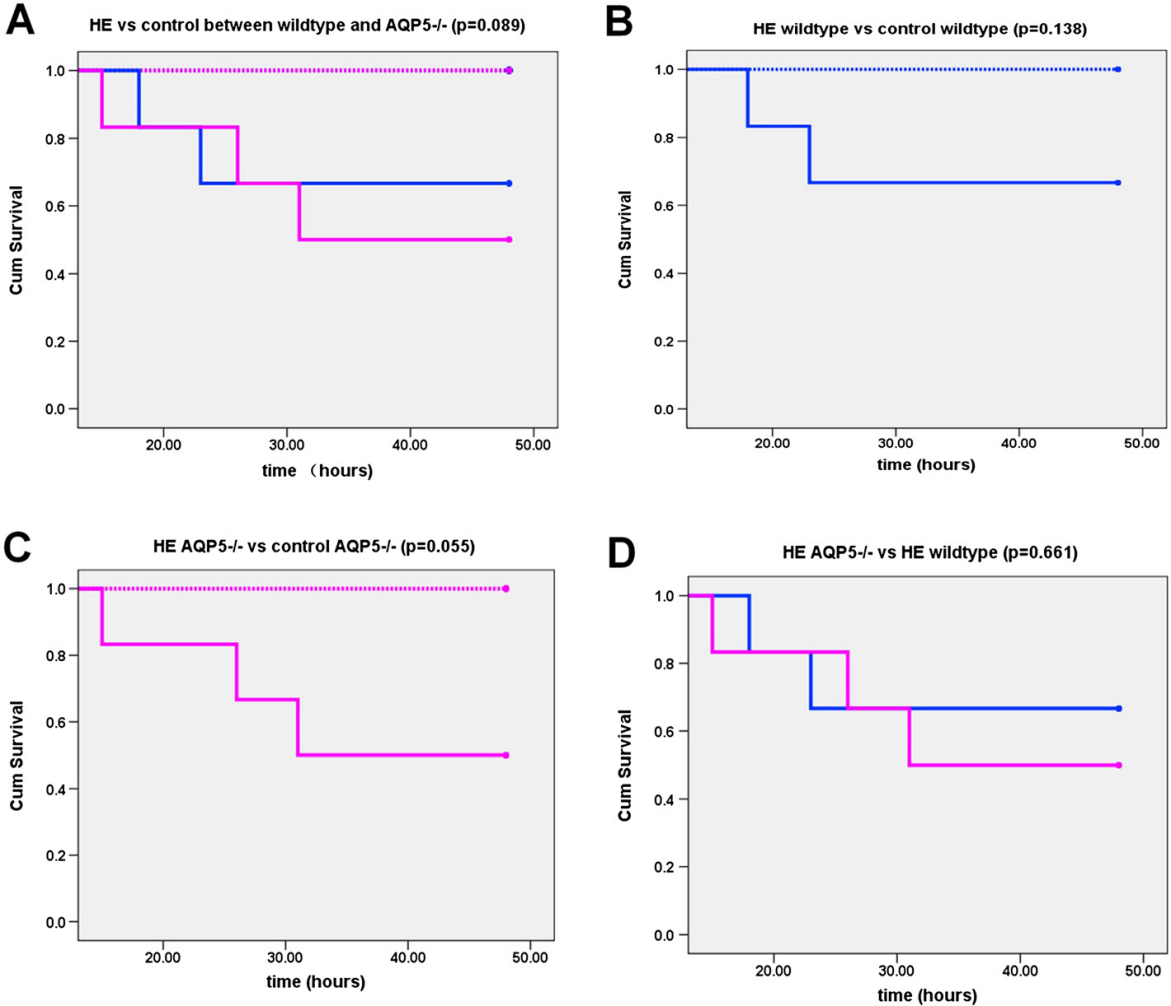
**Survival curve.** There were no significant differences between AQP5 −/− and wildtype *p* > 0.05. **A**: HE vs control between wildtype and AQP5 −/−, **B**: HE wildtype vs control wildtype, **C**: HE AQP5 −/− vs control AQP5 −/−, **D**: HE AQP5 −/− vs HE wildtype. Control wildtype, blue dotted line; Control AQP5 −/−, red dotted line; HE wildtype, blue full line; HE AQP5 −/−, red full line.

Five out of nine mice exposed to hypoxia with exercise died in the 48 hours experiment (three AQP5 −/− mice and two wildtype mice). All animals in the other experiment groups survived (Figure 1).

### Lung wet-to-dry ratio

**Figure 2:**
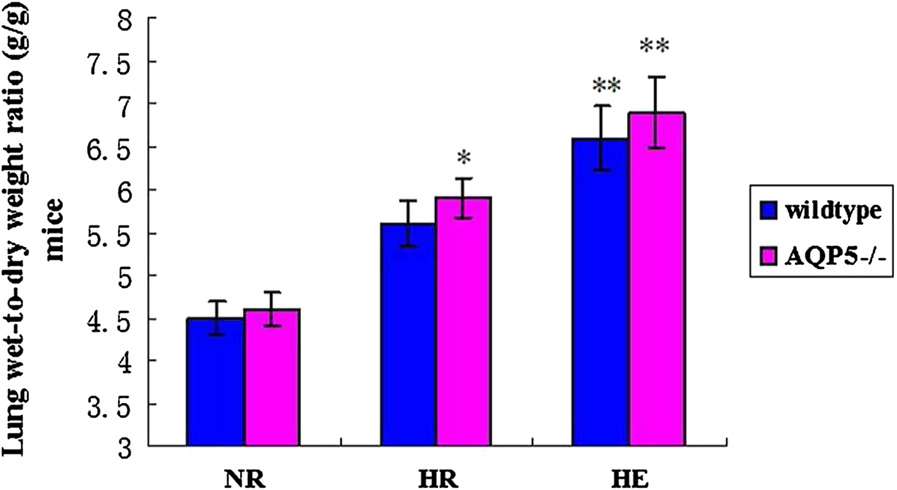
**Lung wet-to-dry weight ratio.** **p < 0.01 for the HE groups between AQP5 −/− and wildtype vs. controls; *p < 0.05 for the HR AQP5 −/− vs. the control. n = 6 in each group. Values are given as the mean ± SEM.

The wet-to-dry lung weight ratio was measured to evaluated fluid accumulation in the lung specimens [29]. Compared with the NR groups, the W/D of AQP5 −/− and wildtype mice in the HE groups mice were significantly increased (*p* < 0.01). In the HR group, the W/D of AQP5 −/− is higher than the NR group (*p* < 0.05). However, there was no significant difference in the lung W/D ratio between the HE and HR groups between AQP5 −/− mice and wildtype mice (Figure 2).

### BAL

**Figure 3:**
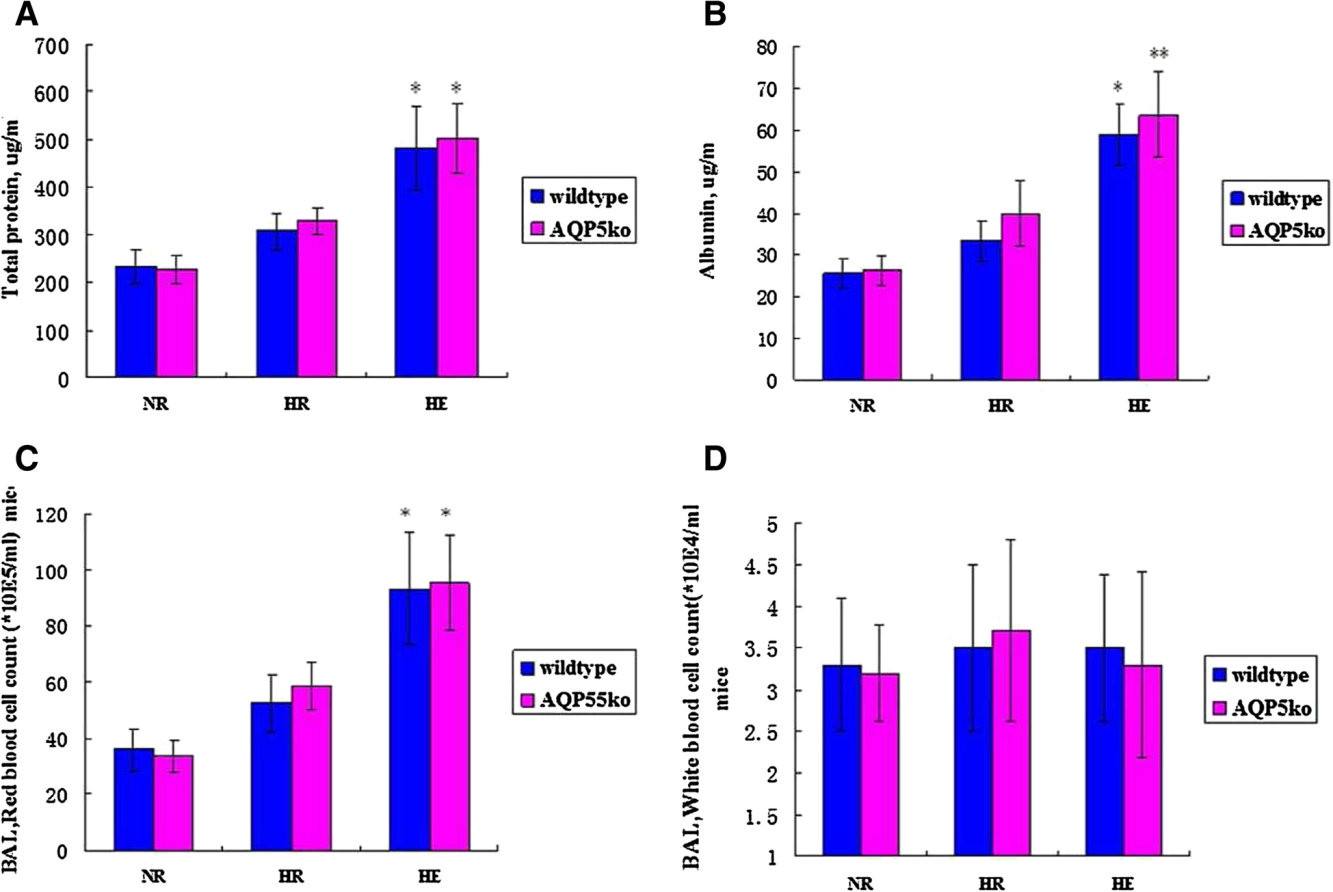
**Protein content and cell counts in BAL. (A)** Total protein. *p < 0.05 for the HE groups between AQP5 −/− and wildtype vs. controls. **(B)** Albumin. **p < 0.01 for the HE AQP5 −/− group vs. controls. *p < 0.05 for the HE wildtype group vs. controls. n = 6 in each group. Values are given as the mean ± SEM. **(C)** Red blood cell counts. * *p* < 0.05 for the HE groups between AQP5 −/− and wildtype vs. controls. **(D)** White blood cell counts. There were no significant differences within these groups. n = 3 in each group. Values are given as the mean ± SEM.

BAL protein is an indicator of lung alveolar-capillary membrane permeability changes [30]. The total protein of HE group both AQP5 −/− mice and wildtype mice were all increased compared with the NR group (*p* < 0.05) (Figure 3A). In addition, the albumin in the HE group has the same results accompany with the total protein, which is higher than the NR group, especially in AQP5 −/− mice (*p* < 0.01) (Figure 3B). However, there was no significant difference between the AQP5 −/− and the wildtype mice of total protein and albumin in the HE group (*p* > 0.05) (Figure 3A, B). At the same time, the alveolar red blood cell counts in AQP5 −/− mice and wildtype mice were all increased in the HE group compared with the NR group (*p* < 0.05, respectively) in BAL (Figure 3C). However, there was no significant difference between the AQP5 −/− mice and wildtype mice in both HE group and NR group (*p* > 0.05) (Figure 3C).

### Lung edema and morphology

**Figure 4:**
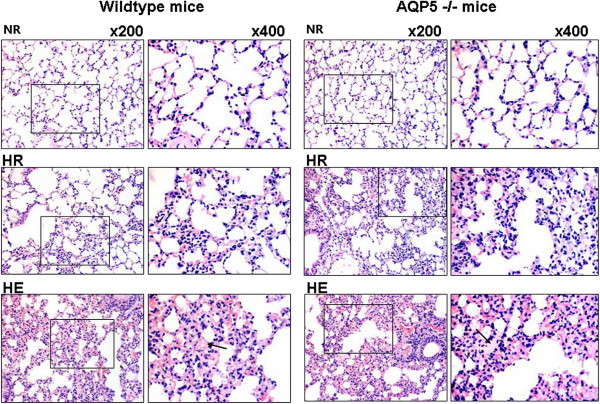
**Lung histology in HAPE mice.** Light micrograph of hematoxylin and eosin stained paraffin embedded section AQP5 −/− and wildtype vs. controls. The alveolar edema was shown by arrows. Magnification × 200 and × 400.

Using light microscopy, we investigated the details of lung morphology in the study. Compared with the NR groups, lung slices form the HE group displayed markedly alveolar oedema, thickening of pulmonary interstitium swollen and red blood cells in the alveolar space (Figure 4). However, there was no significant different between the AQP5 −/− mice and wildtype mice in HE group. In addition, the AQP5 −/− mice in the HR group displayed significantly thickened alveolar septum and few red blood cells in alveolar space, but no obvious alveolar edema than the wildtype mice in the HR group, which suggested that hypoxia *per se* does not induce HAPE (Figure 4).

### Alveolar-capillary permeability

**Figure 5:**
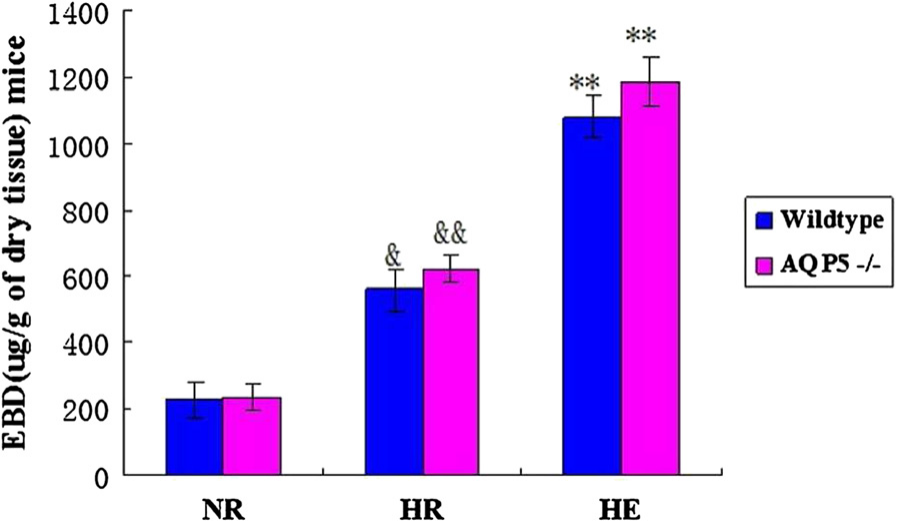
**Alveolar-capillary permeability by Evan’s blue method.** **p < 0.01 for the HE groups between AQP5 −/− and wildtype vs. HR and controls; &&*p* < 0.01 for the HR AQP5 −/− vs. HE and controls; &*p* < 0.05 for the HR wildtype vs. HE and controls. n = 3 in each group. Values are given as the mean ± SEM.

Evan’s blue method was performed to inspect the alveolar-capillary permeability by the extravasation leaked into the lung parenchyma [31]. Compared with the NR and HR group, the EBD of HE group was increased obviously both in the AQP5 −/− mice and wildtype mice (*p* < 0.01, Figure 5). Similarly, EBD in lungs of AQP5 −/− and HR wildtype mice in HR group was significantly higher than NR group respectively (*p* < 0.01, *p* < 0.05,). Moreover, in the HE and HR groups, the lung Evan’s blue leakage of AQP5 −/− mice was slightly increased compare to the wildtype mice but have no significant difference between them (*p >* 0.05) (Figure 5).

## Discussion

Previous results believed that the high-altitude pulmonary edema (HAPE) is a lethal, non-cardiogenic form of pulmonary edema that afflicts susceptible individuals above 3000 m sea level. But a few of people have better tolerant to hypoxia and the development of HAPE [15,32]. According to the previous results, the hypoxia and exercise are considered as the main reasons of HAPE. Therefore, we established mice models of HAPE-like symptoms to investigate the mechanisms of HAPE under the exertion and hypoxic conditions. While the mice walked on treadmill and exposed on hypobaric hypoxia chamber which contribute to the increasing wet-to-dry weight ratio, lung total protein, albumin and blood cell counts in BAL (Figures 2 and 3). These data suggested that the HE group mice developed typical HAPE-like symptoms as determined by physiological analysis but the conditions need to be further optimized. Indeed, although BAL total protein and albumin concentration were obviously increased in mice exposed to hypoxia, the increase was higher when mice were simultaneously exposed to both hypoxia and exercise (Figure 3), suggesting an exacerbating of endothelial permeability [10,33]. In addition, microscopy analysis explored that hypoxia alone induces slight alveolar epithelium and capillary endothelium disruption and swelling, while combined hypoxia and exercise significantly increased endothelium swelling and disruption. In the study, the alveolar-capillary permeability is measured to the integrity of alveolar-capillary barrier by Evan’s blue method [24,27]. Moreover, in our study, HR mice, present the medium situation symptoms that from normal to HAPE development, which could correspond to subclinical HAPE in human. Indeed, the Evan’s blue content, interstitial edema score, the BAL albumin concentration the W/D ratio and the BAL red blood cell content in lungs of HR group were significantly higher compared with NR and HE groups. They were consistent with a significant increase in the lung wet-to-dry weight ratio and bronchoalveolar lavage in the present study. These results suggested Evan’s blue method could be detected the alveolar-capillary permeability for HAPE. Previous results indicated that hypoxic pulmonary vasoconstriction induced pulmonary hypertension in development of HAPE [34]. In addition, the alveolar fluid leaking maybe contribute to, at least in part, the pathogenic mechanism of alveolar epithelium integrity [7,35]. So, the Alveolar epithelium swelling, disruption and alveolar flooding during hypoxia and exercise suggest maintaining epithelium barrier is of vital importance to the maintenance of alveolar-capillary integrity and normal alveolar function during HAPE development.

Another factor that has been believed to play an important role in the pathogenesis of HAPE is inflammation [11]. Previous studies reported that AQP5 deletion in salivary gland inhibits active fluid secretion in the lumen of serous acini [36], which is responsible for the majority of water transport across the apical membrane of type I alveolar epithelial cells [37]. Since AQP5 bi-directionally facilitates fluid transport, defining of the AQP5 effects on lung edema formation and resolution [25,38]. However, there is no direct evidence to prove that the AQP5 maintained alveolar-capillary barrier integrity in HAPE. Thus we compared the depletion of AQP5 mice and wildtype mice in affects lung edema formation and lung histology for HAPE. In this study, the alveolar-capillary permeability, the lung wet-to-dry weight ratio and rich protein in BAL were significantly increased in the AQP5 −/− mice of HE group. And histologic evidence of hemorrhagic pulmonary edema was distinctly shown in HE groups. These results suggest that AQP5 may play, at most in part, an important role in alveolar epithelial barrier function in formation of lung edema involve fluid transport for HAPE.

We addressed the question that AQP5 is located on the alveolar epithelium and thus would not contribute to interstitial compartments across the microvascular endothelium [14]. Previous study show that secretions Surfactant Protein-A (SP-A) are described to useful for the diagnostics of pulmonary edema and it plays a significant role on the human pulmonary disorders and lung diseases [39]. Mechanistically, it is understandable why AQP5 should not facilitate the alveolar-capillary integrity. Further, it is believed that the accumulation of alveolar edema involves transient breakdown the integrity of the alveolar-capillary barrier, permitting the entry of solutes and protein. Therefore, bulk movement of fluid through rifts in the alveolar barrier would not be expected to involve in AQP5. However, our data do not rule out the possibility that AQP5 is involved in other HAPE pathophysilology such as resolution of lung edema [40], gas exchange, alveolar epithelial cell migration and volume regulation [17]. An important function of the epithelium is the active clearance of fluid from the alveolar alveolar-spaces. Alveolar fluid absorption contributes to the resolution of pulmonary edema in HAPE. Water transports is driven by osmotic gradients created by salt transport by a presumed near-isomolar transport mechanism in which the osmotic gradient drive water movement across the highly alveolar-capillary permeability [41]. It is not clear how an apical membrane water-transporting protein might be involved in these processes. Further studies are needed to elucidate these questions.

## Conclusions

In summary, our model of exercising mice exposed to hypoxia is able to induce HAPE-like features. We demonstrated that the high permeability type of HAPE by Evan’s blue method. Maintaining alveolar-epithelium barrier integrity did not appear to be crucial in AQP5 deletion mice, the results indicate that AQP5 plays, at most a part, role in pulmonary edema formation induced by high altitude simulation. The correlation between elevated albumin concentration in the BAL, higher W/D ratio and increased endothelium swelling suggested that alveolocapillary barrier stress failure and the AQP5 played an important role in the development of HAPE. Future research is needed to study the potential function of other aquaporins for HAPE.

## Competing interests

The authors declare that they have no competing interests.

## Authors’ contributions

CXB supervised the conduction of the whole project, and designed JS contributed to the establishment of VILI model in rats, performed the experiments, analysed data and drafted the manuscript; JB and LT did part of the animal study, sample collection and measurement; YLS contributed to revise the manuscript; All authors read and approved the final manuscript.
